# Exploring the influence of health and digital health literacy on quality of life and follow-up compliance in patients with primary non-muscle invasive bladder cancer: a prospective, single-center study

**DOI:** 10.1007/s00345-025-05463-1

**Published:** 2025-01-30

**Authors:** Ahmet Keles, Umit Furkan Somun, Muhammed Kose, Ozgur Arikan, Meftun Culpan, Asif Yildirim

**Affiliations:** School of Medicine, Department of Urology, Istanbul Medeniyet University, Göztepe Prof. Dr. Süleyman Yalçın City Hospital, Fahrettin Kerim Gökay Cd., Istanbul, 34720 Turkey

**Keywords:** Digital health literacy, Patient reported outcome, Bladder cancer, Quality of life

## Abstract

**Objective:**

Given the increasing significance of digital health literacy (DHL) and health literacy (HL) in promoting informed decision-making and healthy behaviors, this study aimed to assess the influence of self-reported HL and DHL on treatment adherence and quality of life among patients who underwent transurethral resection of bladder tumors (TUR-BT) for primary non-muscle invasive bladder cancer (NMIBC).

**Materials & methods:**

This single-center observational study involved patients who underwent TUR-BT for NIMBC at a tertiary hospital from May 2022 to February 2024. Before the procedure, the patients’ DHL and HL were evaluated using the European Health Literacy Survey Questionnaire short version and the eHealth Literacy Scale. Six months after surgery, we surveyed patients’ QoL using the EORTC QLQ-C30. In line with recommendations from the European Association of Urology guidelines, adherence to the treatment plan was assessed along with a follow-up cystoscopy examination for each patient.

**Results:**

Multivariate analysis revealed that poorer DHL and HL were significantly associated with older age (*p* < 0.001), lower educational attainment (*p* < 0.001), and lack of internet access (*p* < 0.001). Conversely, higher DHL and HL levels were positively correlated with increased treatment adherence, as measured by cystoscopy completion (*p* < 0.001). Additionally, logistic regression analysis demonstrated significant associations between improved DHL and HL scores and better global health status (DHL, *p* = 0.022; HL, *p* = 0.008), higher emotional status (*p* < 0.001 for both), and social functioning (*p* < 0.001 for both). Notably, there were no significant differences in the symptom scale scores between the DHL and HL groups.

**Conclusion:**

To the best of our knowledge, this is the first study to explore the specific effect of HL/DHL on QoL and adherence in this patient population. Our research suggests that there may be a link between self-reported levels of DHL/HL and treatment adherence as well as QoL among patients with NIMBC.

## Introduction

Bladder cancer (BC) is a malignancy that is prevalent worldwide, with an estimated 573,000 new cases and 213,000 fatalities annually [1]. Most histological stages of BC are Ta and T1. Although many of these tumors are easy to treat, up to 70% of the patients experience recurrence after surgery [2]. There is a need to educate more than one million people about the treatment options and processes associated with BC. BC patients should play an active role in their treatment decisions and collaborate with healthcare professionals for better outcomes [3]. The only way to achieve this is to educate them properly about cancer, the different treatment options, and the side effects they may experience as a result.

Patients with cancer benefit from social media by receiving health information and emotional support [3, 4]. The internet has become more widely used to access reliable health information [5]. A large amount of online and offline information has led to an infodemic (information pandemic) according to the World Health Organization (WHO) [6]. This presents a significant challenge for cancer patients, as they must navigate this ocean of misinformation while maintaining their trust in the online medical resources they consult. Therefore, patients must be able to comprehend and manage health-related information online. These essential skills are encompassed by digital health literacy (DHL) [5, 7].

Health literacy (HL) refers to the ability to access, understand, evaluate, and apply health information (written or oral) to promote health and care. Low levels of HL are linked to challenges in discussing healthcare-related matters, leading to less effective understanding and implementation of medical advice. It is also associated with difficulties in comprehending written medical information in both clinical and surgical settings as well as reduced adherence to care recommendations and screening protocols [8]. The close relationship between HL and DHL indicates that strategies designed to improve HL can also help enhance DHL.

We hypothesized that providing BC patients with information on disease-related processes, risks, recommendations, and possible situations that may occur after transurethral resection of bladder tumors (TUR-BT) could significantly improve their DHL/HL, thereby helping them cope with the challenges they face and enhance their quality of life (QoL). However, no study has specifically addressed the connection between HL/DHL and QoL in patients receiving TUR-BT for BC. To fill this gap, we conducted this study at a single institution to examine the effects of HL and DHL on QoL and treatment adherence in patients with primary non-muscle invasive bladder cancer (NIMBC) who underwent TUR-BT.

## Materials and methods

### Strategy and ethical principles

This observational study was conducted at a single center and involved patients who had undergone TUR-BT for NIMBC at a tertiary hospital between May 2022 and February 2024. Subsequently, these patients underwent cystoscopic follow-up every three months. The Institutional Review Board of Istanbul Medeniyet University School of Medicine has approved this study (Number: 2022/0324, Date: 18.05.2022). All participants were given a comprehensive overview of the study’s aims and methodologies and were requested to provide written consent. The participants were guaranteed confidentiality of their information, and the study followed the ethical guidelines outlined in the Declaration of Helsinki.

### Selection criteria and data collection

**Fig. 1:**
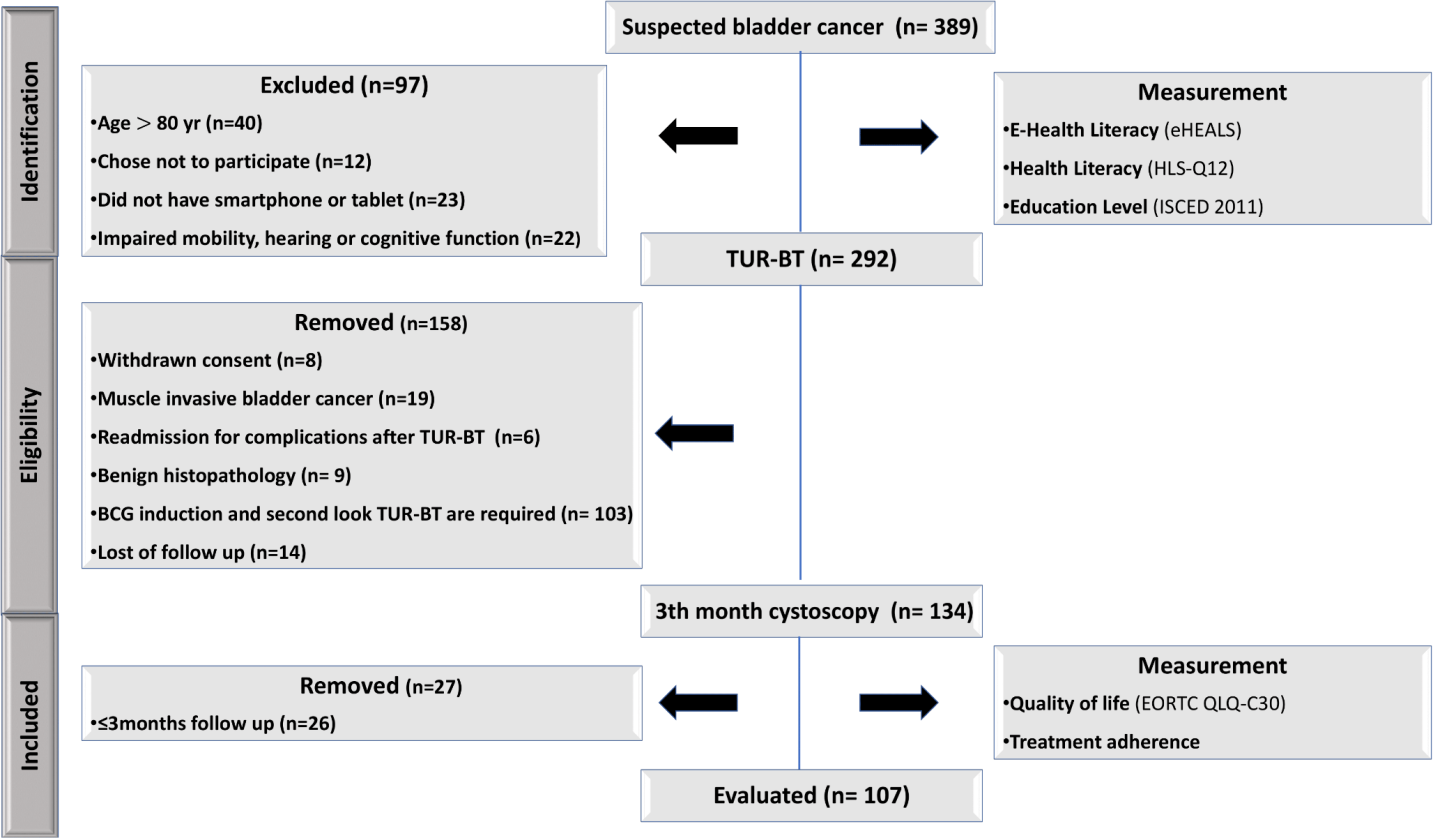
Study Flowchart for Participant Tracking

Figure 1 illustrates the study selection process. Data were collected via in-person interviews in which participants responded to a questionnaire administered by a chef resident. At the beginning of the study, participants were asked various questions to gather sociodemographic data, including age, body mass index (BMI), education level, family income, internet usage, and adherence to cystoscopy.

We included the presence of NIMBC in patients who underwent TUR-BT and subsequent pathological examination. Patients were excluded if they met any of the following criteria: age ≥ 80 years, diagnosis of metastasis, pathological results showing invasive bladder cancer, limited follow-up period (< 6 months), dropouts, cognitive impairments or difficulties in language and comprehension, declined to participate in the study, severe cardiovascular disease, or advanced respiratory illness. Additionally, considering that the complexity of regimen maintenance, BCG treatment, and second-look TUR could affect QoL, we excluded these patients from the study.

### Defining the instruments and measurement

This eight-item e-Health Literacy Scale (e-HEALS) exam assesses consumers’ capacity to discover, analyze, and apply electronic health information to their health issues. Each feature of the measure was evaluated using a Likert scale ranging from one to five. Three question scores were added to obtain a DHL score. The scale values vary from 8 to 40, with high scores indicating high DHL [7].

The HLS-Q12, a validated and abridged version of the European Health Literacy Survey Questionnaire, was used to determine the HL levels. It comprises 12 questions intended to assess a person’s confidence and competence in handling various health circumstances. Patients rated the complexity of each circumstance on a four-point Likert scale, from simple to complex, with a higher score signifying higher HL proficiency [8, 9].

The Turkish version of the European Organization for Research and Treatment of Cancer (EORTC) QoL Questionnaire (QLQ)-C30 was administered six months after TUR-BT. The questionnaire consisted of 30 questions divided into five functional dimensions: physical, role, emotional, cognitive, and social. There were also three symptom measures (fatigue, pain, and nausea/vomiting) and a general health status section. The six sections address various symptoms, including shortness of breath, loss of appetite, sleep difficulties, constipation, diarrhea, and financial concerns [10]. All surveys were examined in accordance with their respective scoring guidelines. The BMI was divided into three groups based on WHO criteria [11].

Educational attainment was classified according to the International Standard Classification of Education (ISCED) of the United Nations Educational, Scientific, and Cultural Organization [12]. The 2023 governmental mandate sets a national minimum wage of $10,000 based on income classification.

### Treatment compliance

After discharge, patients were assessed in the outpatient clinic in their third month for routine cystoscopy follow-up, according to the European Association of Urology guidelines. Patients were classified into two groups according to their adherence to the cystoscopy follow-up protocol: compliant and non-compliant. Each patient who did not receive the cystoscopic follow-up was identified: as “non-compliant”. We relaxed the definition to include compliance with cystoscopic follow-up at any time within 90–120 days of TUR-BT.

### Statistical analysis

We used the average and standard deviation to analyze continuous variables, and percentages to assess categorical variables. The normal distribution of continuous variables was assessed using the Shapiro-Wilk and Kolmogorov-Smirnov tests. One-way ANOVA was used to analyze differences between groups in continuous variables. To examine multiple continuous variables, a multivariate analysis of variance was used. Statistical analysis of continuous variables included the application of Mann–Whitney U and Kruskal–Wallis tests, with statistical significance set at *p* < 0.05.

## Results

### Patient demographics

The final analysis included data from 107 participants with Ta disease: 56.1% (*n* = 60) had low-grade tumors, 23.4% (*n* = 25) had focal high-grade tumors, and 20.6% (*n* = 22) had high-grade tumors. The median age at surgery was 67.5 years (range: 40–79 years), and there were 92 males and 15 females with a median BMI of 25.3 kg/m² (range: 21.6–32.3 kg/m²). Marital status assessment revealed that 84.1% of the participants were married, while the remaining 15.9% were single. Baseline HL scores averaged 33.4 ± 2.9, and DHL scores averaged 24.5 ± 1.7.

### Factors associated with health literacy and digital health literacy

Individuals with lower HL and DHL scores were more likely to be older (*p* = 0.006 for HL, *p* = 0.014 for DHL) and have lower educational attainment (*p* < 0.001). Additionally, lower income and lack of internet access were negatively correlated with both HL and DHL scores (*p* = 0.011 for HL and *p* < 0.001 for DHL). To understand the factors influencing HL and DHL scores, the study includes Table 1, outlining the results of univariate and multivariate analyses.

**Table 1:** Univariate and multivariate analyses to identify the factors that impact health literacy and digital health literacy scores

Variables		*N* = 107 (%)	Health Literacy		Digital Health Literacy		Multivariate analysis p value**
			Mean ± SD	p value*	Mean ± SD	p value*	
Age (yr.)	≤ 59	17 (15.9)	37 ± 4.31	**0.006**	29.21 ± 6.31	**0.014**	**< 0.001**
	60–69	63 (58.9)	33.53 ± 3.64		24.27 ± 6.74		
	≥ 70	23 (32.7)	29.22 ± 4.3		18.31 ± 7.12		
BMI (kg/m2)	Normal (< 25)	29 (27.1)	32.68 ± 6.32	0.591	22.61 ± 6.26	0.679	0.732
	Overweight (25–30)	56 (52.3)	31.43 ± 6.31		22.77 ± 6.21		
	Obese (≥ 30)	22 (20.6)	33.68 ± 4.51		23.81 ± 5.52		
Education level (ISCED level)	Level 1	42 (39.2)	28.11 ± 5.63	**0.007**	18.31 ± 6.98	**< 0.001**	**< 0.001**
	Level 2	28 (26.2)	30.6 ± 5.41		24.73 ± 5.43		
	Level 3	23 (21.5)	33.541 ± 4.71		26.81 ± 4.1		
	Level 4	14 (13.1)	37.3 ± 4.53		30.63 ± 5.39		
Marital Status	Single	17 (15.9)	31.35 ± 4.24	0.214	21.12 ± 6.36	0.768	0.945
	Married	90 (84.1)	32.03 ± 5.32		21.88 ± 4.97		
Smoking Status	Yes	75 (70.1)	32.8 ± 5.44	0.873	21.59 ± 3.62	0.822	0.868
	No	14 (13.1)	31.83 ± 4.49		21.37 ± 6.41		
	Ex-smoker	18 (16.8)	32.18 ± 3.21		20.68 ± 4.74		
Annual income ($)	≤ 10.000	75 (70.1)	30.47 ± 3.87	**< 0.001**	20.17 ± 6.19	**< 0.001**	**< 0.001**
	> 10.000	32 (29.9)	36.44 ± 0.73		30.33 ± 4.73		
Internet usage	Almost everyday	29 (27.1)	33 ± 4.2	**0.011**	30.63 ± 5.95	**< 0.001**	**< 0.001**
	Few days a week	36 (33.6)	32.51 ± 6.3		25.74 ± 3.91		
	Less than 1 day a week	16 (15)	29.77 ± 5.31		23.5 ± 6.72		
	Hardly ever	26 (24.3)	29.13 ± 5.81		17.2 ± 5.1		
Cystoscopy adherence	Compliance	74 (69.1)	33.21 ± 6.18	**0.021**	26.39 ± 5.88	**< 0.001**	**< 0.001**
	Non-Compliance	33 (30.9)	29.46 ± 5.44		21.42 ± 6.27		

*ANOVA. **MANOVA (Wilk’s Lambda); yr. = years; BMI = Body mass index; ISCED = International Standard Classification of Education; Bold font indicates statistically significance

### Quality of life outcomes

Table 2 presents a comparison of QoL evaluations derived from the EORTC QLQ-C30 for both the HL and DHL groups. Limited HL and DHL were correlated with lower global health scores than moderate or advanced HL/DHL (*p* = 0.022 and *p* = 0.008, respectively). The patients in the low HL/DHL group also faced more financial difficulties (*p* = 0.032 and *p* = 0.043, respectively). Logistic regression analysis indicated a significant positive correlation between elevated HL and DHL levels and global health (*p* = 0.023 and *p* = 0.021, respectively). Furthermore, individuals with higher DHL and HL scores reported improved emotional and social functioning (both *p* < 0.001). However, no statistically significant differences were observed in the symptom scale scores across the DHL groups (Table 3).

**Table 2:** Investigating the levels of patients’ health literacy and digital health literacy and their relationship to the EORTC QoL subscale scores

Endpoint	Health Literacy				Digital Health Literacy		
EORTC QLQ-C30	Limited HL (Score ≤ 26)	Moderate HL (27 ≤ Score ≤ 39)	Advanced HL (Score ≥ 39)	*p* value*	Low DHL, (Score ≤ 24)	High DHL, (Score >24)	*p* value **
	Mean ± SD	Mean ± SD	Mean ± SD		Mean ± SD	Mean ± SD	
Global health status / QoL^†^							
Global Health Status (Q29,30)	58.43 ± 15.05	69.16 ± 25.64	81.5 ± 13.86	**0.022**	61.31 ± 15.44	75.39 ± 25.48	**0.008**
Functional Scales^†^							
Physical Function (Q1 to 5)	66.3 ± 20.64	74.06 ± 20.85	62.5 ± 25.68	0.104	66.14 ± 17.66	66.70 ± 13.82	0.653
Role Function (Q6,7)	71.82 ± 18.13	69.77 ± 24.51	72.4 ± 19.92	0.902	61.42 ± 21.27	60.29 ± 20.46	0.702
Emotional Function (Q21 to 24)	59.43 ± 15.5	66.38 ± 20.92	85.28 ± 15.92	**0.017**	60.62 ± 19.31	81.08 ± 24.63	**0.026**
Cognitive Function (Q20,25)	75.93 ± 19.34	74.06 ± 20.85	60.00 ± 16.04	0.066	74.72 ± 20.91	71.77 ± 20.20	0.320
Social Function (Q26,27)	64.69 ± 14.19	66.38 ± 20.92	70.83 ± 26.35	0.734	76.73 ± 20.5	62.65 ± 21.37	0.099
Symptom Scales^‡^							
Pain (Q9,19)	42.55 ± 19.37	41.07 ± 23.60	35.57 ± 23.29	0.321	33.65 ± 23.68	43.46 ± 27.3	0.074
Nausea and vomiting (Q14,15)	10.31 ± 21.02	12.56 ± 18.09	11.25 ± 13.29	0.745	13.84 ± 22.82	13.07 ± 17.1	0.652
Fatigue (Q10,12,18)	43.05 ± 15.11	34.97 ± 20.36	34.89 ± 26.56	0.311	34.59 ± 21.86	38.34 ± 22.15	0.42
Dyspnoea (Q8)	30.53 ± 21.12	20.25 ± 10.67	15.60 ± 9.77	0.284	17.61 ± 15.82	21.57 ± 19.8	0.13
Insomnia (Q11)	34.88 ± 18.87	25.83 ± 26.51	37.5 ± 37.53	0.424	28.3 ± 28.79	28.10 ± 26.14	0.939
Appetite loss (Q13)	38.75 ± 20.97	25 ± 26.25	23.33 ± 25.2	0.365	26.41 ± 29.5	22.87 ± 20.54	0.912
Constipation (Q16)	26.25 ± 21.56	25 ± 24.59	25 ± 38.83	0.623	28.30 ± 25.65	22.87 ± 25.38	0.24
Diarrhea (Q17)	18.75 ± 15	11.67 ± 19.92	8.33 ± 15.43	0.061	15.09 ± 19.13	10.46 ± 19.43	0.122
Financial difficulties (Q28)	22.42 ± 14.36	19.95 ± 21.33	17.85 ± 22.32	**0.032**	29.9 ± 23	23.53 ± 22.4	**0.043**

**EORTC QLQ-C30**: European organization for research and treatment of cancer core quality of life questionnaire; *Kruskal Wallis Test; **Mann Whitney U Test; **†** Higher scores indicate better functioning (scaled from 0-100); **‡**Lower scores indicate fewer symptoms (scaled from 0-100)

**Table 3:** Comparison of patients’ health literacy and electronic health literacy classifications and EORTC QoL subscale scores by multivariate analysis

EORTC QLQ-C30	Health Literacy ^¥^		Digital Health Literacy	
	OR (95% CI)*	*p* value*	OR (95% CI)*	*p* value*
Global health status / QoL				
General Health Status (GHS)a	0.95 (0.83–2.97)	**0.023**	1.99 (0.96–3.03)	**0.021**
Functional Scales				
Physical Function (Q1 to 5)a	1.05 (0.81–1.33)	0.109	1.06 (0.94–1.92)	0.653
Role Function (Q6,7)a	1.52 (0.95–2.41)	0.042	1.34 (0.94–1.80)	0.129
Emotional Function (Q21 to 24)a	3.43 (2.69–5.99)	**< 0.001**	3.94 (2.73–5.65)	**< 0.001**
Cognitive Function (Q20,25)a	0.98 (0.83–1.65)	0.859	2.98 (2.95–4.62)	0.878
Social Function (Q26,27)a	1.06 (0.93–1.87)	**< 0.001**	0.93 (0.75–1.11)	**< 0.001**
Symptom Scales				
Pain (Q9,19)b	1.41 (0.76–1.91)	0.94	1.02 (0.94–1.11)	0.132
Nausea and vomiting (Q14,15)b	1.65 (0.98–1.89)	0.321	0.97 (0.85–1.37)	0.953
Fatigue (Q10,12,18)b	0.81 (0.72–1.69)	0.095	0.96 (0.92–1.23)	0.789
Dyspnoea (Q8)b	1.07 (0.89–1.19)	0.112	1.52 (0.99–1.95)	0.871
Insomnia (Q11)b	0.8 (0.76–1.12)	0.19	1.38 (0.5–1.81)	0.574
Appetite loss (Q13)b	1.08 (0.92–1.21)	0.231	3.96 (0.93–4.46)	0.134
Constipation (Q16)b	1.02 (0.97–1.21)	0.481	2.95 (0.96–5.09)	0.653
Diarrhea (Q17)b	0.99 (0.89–1.06)	0.545	0.94 (0.9–1.08)	0.321
Financial difficulties (Q28)b	1.01 (0.93–1.12)	**0.012**	1.98 (0.95–2.02)	**0.042**

OR = Odds ratio; CI = confidence interval; SD = Standard deviation; Q = question; GHS = global health status; QoL = quality of life; a range = 0–100, high values indicate high levels of functioning and quality of life; b range = 0–100, high levels indicate pronounced symptoms and problems.; ^¥^HL was classified as nonadequate (limited) and adequate (moderate-advanced) *Binary Logistic Regression-Enter method was used. Age group, educational level, internet usage status, HL and EORTC subscales was added to model

### Treatment adherence

Among the 107 participants, 74 (69.1%) adhered to the recommended cystoscopy follow-up protocol, whereas 33 (18%) did not. A positive correlation emerged between higher HL/DHL scores and improved treatment adherence, as evidenced by increased cystoscopy completion rates (*p* < 0.001).

### Follow-up and healthcare utilization

Logistic regression was employed to investigate the link between limited and high-level HL/DHL and healthcare utilization within 30 days after surgery. The research uncovered no link between low HL/DHL and increased emergency department visits (*p* = 0.625, CI: 0.74–1.82) or urology outpatient clinic readmissions (*p* = 0.821, CI: 0.88–1.4).

## Discussion

The presentation of this second study, which is consistent with our previous study in which HL and DHL were first demonstrated to be effective in urological oncology, opens a new avenue for discussion in patients with low-risk NMIBC [13]. Our research has yielded valuable insights into the relationship between HL/DHL levels in patients with NIMBC, their health-related QoL, and compliance with treatment.

Our results indicate that there is a correlation between lower HL/DHL and advancing age, lower levels of education, and limited internet accessibility. The decrease in HL and DHL levels with increasing age could be explained by the fact that, as individuals age, their body functions tend to decline, which can lead to a reduction in the capacity to process and retain new information, ultimately impacting HL/DHL scores. The relationship between older age and hearing loss is especially notable in the context of BC. This is due to the fact that 80% of bladder cancer diagnoses occur in individuals over the age of 65 [14, 15].

Numerous studies have found a strong link between educational level and HL/DHL scores. Higher levels of education are linked to improved capacity in locating, assessing, and utilizing health-related information for decision-making [16]. Furthermore, lack of Internet access is a major barrier to DHL, as it limits access to a wide range of online health information and resources [17, 18]. Together, these insights highlight the pressing need for targeted interventions and educational initiatives to enhance DHL and HL among vulnerable patients with BC.

Our study revealed that patients with lower HL/DHL experienced more challenges in understanding information, reported worse emotional and social functioning outcomes, and had a lower QoL, whereas those with higher HL/DHL appeared to be better informed and had a higher QoL. Xia et al. discovered that cancer survivors with sufficient health literacy were almost three times more likely to experience a higher QoL compared to those with inadequate HL [19]. The researchers also noted similar findings in other measures of functional well-being.

A variety of interventions, including information handouts, audiovisual materials, and online resources, can help improve patients’ HL/DHL and treatment adherence [20, 21]. These interventions not only facilitate better understanding of medical information but also empower patients to actively participate in their own care. Patients with enhanced HL/DHL are more inclined to comply with follow-up schedules and treatment plans, thereby contributing to improved overall health results.

Furthermore, disease progression and relapse play significant roles in BC prognosis. Recurrence rates within 5 years range between 30% and 80%, while progression rates vary from 1 to 45% [22]. Our findings suggest that lower levels of HL and DHL are associated with poorer health-related treatment adherence among NMIBC patients, consistent with a study by Turkoglu et al. Patients with low HL might not possess the necessary numerical abilities to comprehend and apply information about the probability of BC recurrence or the likelihood of cancer remission [23]. As the prognosis of those who fully adhere to the follow-up protocol is expected to be better, informing BC patients with low HL with additional written and visual materials may be a cost-effective and progressive preventive approach [24, 25].

While the study possesses strengths, acknowledging its limitations is crucial. Firstly, we were unable to determine pre-operative diagnoses of generalized anxiety disorder, clinical depression and baseline QoL. Secondly, the study did not consider other potential factors influencing the outcome of the TUR-BT procedure, such as patient performance or frailty status. Thirdly, the sample size was relatively small, and participants belonged to a specific demographic group limiting the applicability of the results. Finally, the accuracy of the questionnaires relied on patient honesty and cooperation, which represents a significant limitation. However, to the best of our understanding, this is the initial investigation focusing on how HL/DHL affects the QoL and adherence of NIMBC patients. In addition, established and validated questionnaires were used in this pioneering study. Future research efforts should address these limitations by employing larger, more diverse patient populations and incorporating additional factors that might influence treatment outcomes.

## Conclusion

The findings underscore the need for healthcare providers to assess and address HL and DHL in their patient populations to deliver more effective and patient-centered care. The idea of including HL/DHL in every aspect of BC care by all healthcare providers is important for providing sufficient support and the best possible information to BC patients with different levels of HL/DHL. This will ultimately improve patient satisfaction, QoL, and adherence to treatment plans. It will also contribute to a more holistic approach to patient care.

## Data Availability

No datasets were generated or analysed during the current study.
